# Shifting sands—Molecular coronavirus testing during a time of inconsistent resources

**DOI:** 10.1017/ice.2020.275

**Published:** 2020-06-05

**Authors:** David K. Henderson, Mary K. Hayden, Sharon B. Wright, David J. Weber, A. Rekha Murthy, Hilary Babcock, Judith A. Guzman-Cottrill, Sarah Haessler, Clare Rock, Trevor Van Schooneveld, Corey A. Forde, Latania K. Logan, Anurag Malani

**Affiliations:** 1 National Institutes of Health, Bethesda, Maryland; 2 Rush University Medical Center, Chicago, Illinois; 3 Division of Infection Control/Hospital Epidemiology, Beth Israel Deaconess Medical Center, Boston, Massachusetts; 4 Hospital Epidemiology, University of North Carolina Health Care, Chapel Hill, North Carolina; 5 Division of Infectious Diseases, UNC School of Medicine, Chapel Hill, North Carolina; 6 Department of Medical Affairs, Cedars-Sinai Medical Center, Los Angeles, California; 7 Washington University School of Medicine, St Louis, Missouri; 8 Division of Infectious Diseases, Department of Pediatrics, Oregon Health and Science University, Portland, Oregon; 9 Baystate Health, Springfield, Massachusetts; 10 Division of Infectious Diseases, Department of Medicine, Johns Hopkins University School of Medicine, Baltimore, Maryland; 11 Division of Infectious Disease, Department of Internal Medicine, University of Nebraska Medical Center, Omaha, Nebraska; 12 Queen Elizabeth Hospital, Barbados; 13 Division of Infectious Diseases, Department of Internal Medicine, Department of Infection Prevention & Control, St Joseph Mercy Health System, Ann Arbor, Michigan

***Editor’s Note***: This is the first in a series of single-topic State-of-the-Pandemic commentaries from the SHEA Board of Trustees. These short commentaries are designed to provide perspective for institutions facing challenges at various stages of the pandemic. Subsequent commentaries will address ongoing controversies, creative solutions to challenging problems, and strategies designed to help healthcare epidemiology advance in the face of the pandemic to provide the safest care possible for our patients and the safest possible environment for our healthcare provider colleagues.

Virtually everyone agrees that expanded SARS-CoV-2 diagnostic testing using a molecular method will benefit both society in general and healthcare in particular. Expanded testing does, however, require careful thought about its application. Clearly, testing is not a panacea that eliminates risk or prevents the patient from developing infection a few days later. Nonetheless, testing should always be coupled with rigorous infection control practices and clinical vigilance. Whereas testing is rapidly becoming more accessible, testing is not uniformly accessible throughout the nation; it is, unfortunately, less available in both urban and rural settings where it is often needed most. Here, we present a rational approach to testing in settings faced with inconsistent resources. Our intent is to try to identify the best use of existing resources. Virtually all COVID-19 test kits (and the reagents needed to support them) are in short supply, and manufacturers of some of the tests are currently forced to allocate reagents to try to preserve equitable distribution. When testing first became available, it was used almost exclusively for patients who had symptoms consistent with COVID-19 disease. As we have learned about the significance of presymptomatic and asymptomatic spread of the disease, scientists and facility administrators have become interested in testing asymptomatic populations (eg, patients about to be admitted to hospitals, nursing homes, or other congregate settings, contacts of infected patients, surveillance of infection among hospital staff). The purpose of this short paper is to emphasize a few principles that SHEA members are following during these extremely challenging times for healthcare epidemiology, for healthcare in general, and for our nation as a whole. Our intent is to inform epidemiologists, clinicians, and other health professionals about our approach to these complex issues.

1. In settings in which testing resources remain limited, SARS-CoV-2 testing should still be primarily focused on identifying symptomatic patients with COVID-19.
2. One should be mindful that for institutions using laboratory developed tests, reagents are often in short supply, as are test swabs and transport media. These shortages necessitate stewardship of important resources.
3. For commercial assays approved by the FDA under emergency use authorization (EUA), not all specimen sources (eg, bronchoalveolar lavage fluid) are assessed or approved. Therefore, clinical laboratories must do in-house validation of these sources. Similarly, clinical laboratories that developed their own assays for detection of SARS-CoV-2 must validate each specimen source before that source can be tested. In lieu of in-house validation, clinical laboratories may choose to send specimens to a reference laboratory that has validated the specimen source. Turnaround time for the 2 settings (ie, the in-house laboratory vs the reference laboratory) may be quite different; the in-house laboratory can usually turn a test around in 1–48 hours, depending on the exact test used, whereas most commercial laboratory turnaround times range between 48 and 72 hours or more (if transportation time to the reference laboratory is included). Thus, even if a specific sample provides increased sensitivity (eg, lower respiratory tract samples are much more sensitive than upper respiratory tract samples), physicians should consider the importance of the turnaround time. For patients with pneumonic processes, if the turnaround time for both lower and upper respiratory samples are similar, lower respiratory sampling is preferable because of its increased sensitivity. If the lower respiratory specimen result will be delayed, obtaining a nasopharyngeal sample as well (assuming a faster turnaround time) is advisable.
4. Similarly, tests available under an EUA may have different performance characteristics, such as sensitivity and specificity, and to date, we lack a reference standard against which to compare test results. In a recent study (not yet peer reviewed), the sensitivity of a rapid point-of-care test was 67%. The performance characteristics of each test in use and the importance of rapid results must be weighed carefully in designing and implementing test utilization strategies for different settings and situations.
5. Although traditionally a nasopharyngeal swab has been the diagnostic specimen of choice for upper respiratory pathogens, a literature is developing that suggests that, for SARS-CoV-2 detection, other less intrusive approaches (eg, oropharyngeal swabs, saliva, or saliva plus anterior nares swab) may have similar sensitivities. Several centers have already been able to validate such other sources as reliable. If additional data confirm the equivalent sensitivity, institutions will likely validate these new testing approaches because adherence to repeat testing protocols will likely improve with these less intrusive techniques.
6. Because of the need for stewardship of testing resources, a cautious approach to testing asymptomatic individuals (eg, individuals directly exposed, family contacts, etc) is advisable. For example, one could make a strong case for testing asymptomatic individuals in outbreaks or clusters, particularly in high-prevalence congregate settings. When testing is made more available, testing of asymptomatic contacts will almost certainly become more common with 3 likely goals: (1) to try to avoid adverse outcomes in the person being screened, (2) to reduce the exposure of other people to COVID-19, and (3) to guide the appropriate use of PPE by healthcare providers. Conversely, waiting on test results for an asymptomatic patient to decide about care may actually increase the patient’s risk for adverse outcomes. We also emphasize that appropriate timing of postexposure testing still needs to be determined. Testing too early may miss cases that will develop later in the incubation period, providing a false sense of security to the tested individual.
7. Specifically, regarding preprocedure testing, if the providers involved are consistently going to use full PPE (either N95 plus eye goggles or face shield, or a powered air purifying respirator), testing may be of limited immediate value. One could mount an argument for doing preprocedure testing if the result from the test contributed to patient safety or contributed to PPE stewardship. Many professional societies have recommended testing for myriad types of procedures performed by their memberships. Many of these recommendations are made out of an abundance of caution, and their implementation should be assessed in the context of the availability of PPE. Preprocedure testing is most useful if used to guide PPE use by providers and to delay elective procedures on infected patients.

